# Critical Ischemia Following Hyaluronic Acid Filler Injection: A Case Report

**DOI:** 10.3390/jcm14030802

**Published:** 2025-01-26

**Authors:** Jakub Filip Turcza, Joanna Bartosinska, Dorota Raczkiewicz

**Affiliations:** 1 Department of Cosmetology and Aesthetic Medicine, Medical University of Lublin, 20-093 Lublin, Poland; jbartosinski@gmail.com; 2 Department of Medical Statistics, School of Public Health, Centre of Postgraduate Medical Education, 01-813 Warsaw, Poland; dorota.raczkiewicz@cmkp.edu.pl

**Keywords:** critical ischemia, ischemia, hyaluronic acid, filler, skin, aesthetic medicine

## Abstract

**Background:** Vascular complications, following the application of non-permanent, hyaluronic acid-based tissue fillers are a rare but very serious and rapidly progressive disorder that can, in extreme cases, lead to skin necrosis, blindness, or a stroke. Interest in aesthetic procedures is constantly growing, so awareness and knowledge of the correct and comprehensive treatment of complications are important. The human face is an area characterised by complex innervation and vascularisation. There are high-risk areas in which the application of fillers should be carried out with particular care using appropriate techniques and instruments, as well as preparations with specific rheology. The aetiopathogenesis of vascular complications is complex—involving partial or complete vessel occlusion, the presence of local inflammation in the affected tissues, and potential arteriospasm of the vessels supplying the area, resulting in tissue dysperfusion and ischaemia. **Methods:** In this article, the case of a patient who developed signs of a vascular compromise on the forehead area after improperly administering filler in the glabellar and nasal areas is presented. **Result:** The diagnostic and therapeutic management applied, including above all high doses of hyaluronidase, Doppler ultrasound diagnostics, and general medications, as well as a complementary treatment of the distant effects of the complication, i.e., erythema and tissue loss in the forehead area, by autologous injection procedures and laser therapy, resulted in a full recovery and a very good aesthetic result. **Conclusions:** This case proves that complications after aesthetic medicine procedures, including vascular complications are possible. Education of patients and doctors, proper diagnosis, and initiation of appropriate treatment at an early stage of the problem can bring very good therapeutic results for the patient.

## 1. Introduction

Hyaluronic acid-based fillers are non-permanent injectable materials used for soft-tissue augmentation and are one of the basic and essential tools in aesthetic medicine. They allow both fine wrinkles and deep tissue defects, resulting from age or other reasons, to be corrected as well as changing the shape or contouring in specific areas of the face and the entire body. According to data from the American Society for Aesthetic Plastic Surgery (ASAPS), the number of filler treatments in the USA in 2016 was 2.7 million, more than half of which was hyaluronic acid (HA)-based fillers, a marked increase from the 2000 figure of just 600,000 [1,2]. The gradual development of knowledge of filler administration techniques, as well as the increased availability of preparations with different rheologies and physicochemical profiles, makes it possible to select an appropriate personalised treatment, dedicated to the specific area and problem with which the patient comes to the procedure, resulting in an increased safety profile for the procedure.

Complications after non-permanent filler administration are not frequent, but they still occur, carrying an especially elevated risk if the procedure is performed by a non-qualified individual. Typically, such complications are of a vascular nature, the pathomechanism of which is complex and relates to disturbances in vascular blood flow as a result of pressure on the vessel and/or its partial or complete occlusion with filling material (microembolism), as well as the contraction of the vessel walls. If left untreated, this vascular complication leads to persistent tissue perfusion abnormalities, necrosis, and, in extreme cases, permanent vision loss or stroke [2,3,4,5].

Some sources state that up to 3 out of every 1000 treatments with a hyaluronic acid-based filler may occur with vascular disorders, so the application of hyaluronic acid-based fillers in the facial area is a major challenge for the practitioner and requires a thorough knowledge of the anatomy, vascularization, and innervation of the region [1,6].

Some of the high-risk areas where fillers are applied include the glabella, temples, nose, eye area (usually tear trough), nasolabial folds, and upper lip. The appearance of any symptoms that may indicate complications requires the clinician to observe and implement appropriate measures as soon as possible after the appearance of disturbing symptoms [4,7,8,9].

In this article, the case of a patient who developed signs of a vascular compromise after filling superficial forehead wrinkles, nasolabial folds, and nose with an HA-based filler by a beauty service technician is presented.

## 2. Case Report

**Figure 1:**
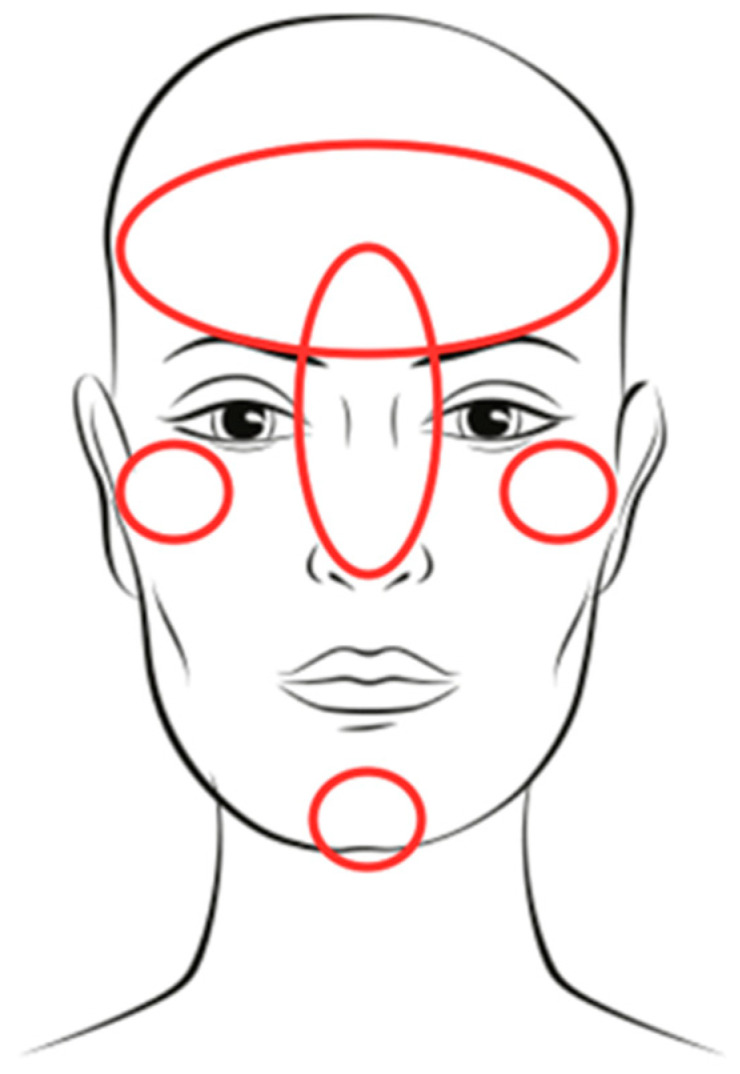
Patient’s facial areas where filler was injected.

A 33-year-old patient underwent blanching of small forehead wrinkles, as well as the filling of the labella wrinkle (“lion’s wrinkle”), nose, zygomatic bones, and chin (Figure 1) with hyaluronic acid filler (Revolax Deep, Chuncheon-si, Gangwon-do, Republic of Korea), at home, by a beautician. According to the patient’s report, a total of 1 mL of HA filler was administered with a needle and there were no disturbing symptoms immediately after the procedure.

The day after the procedure, the patient noticed redness and swelling in the right glabella and central forehead, and over the following 2 days, there were increasing skin changes such as erythema, swelling, and tenderness of the treated area.

**Figure 2:**
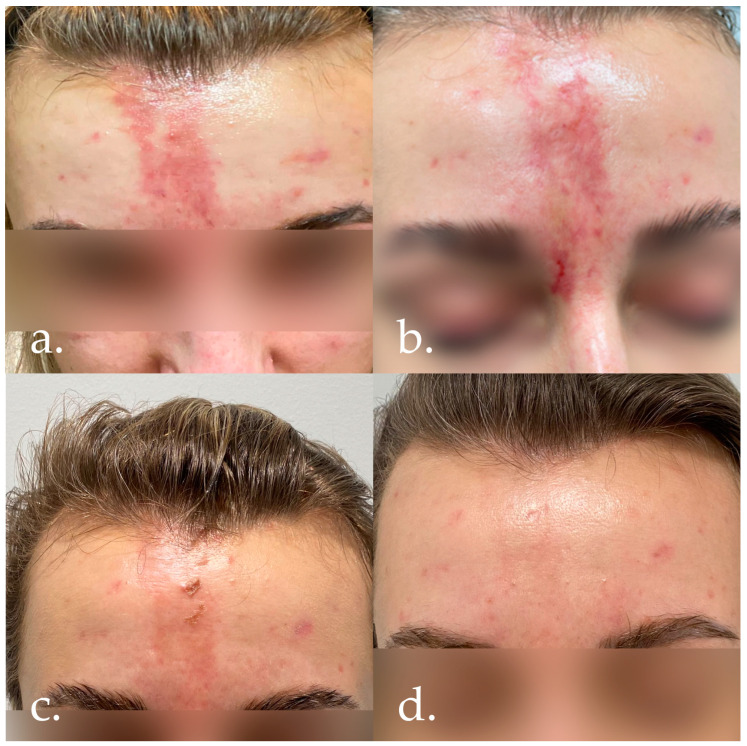
(**a**) Four days after the administration of filler. (**b**) Six days after the administration of filler/1 day after the administration of hyaluronidase. (**c**) Twelve days after the administration of filler/7 days after the administration of hyaluronidase. (**d**) One and a half months after the administration of filler.

Four days after the procedure, the symptoms were further exacerbated (Figure 2a) and the patient discussed the problem with the person performing the procedure. An unknown amount of hyaluronidase was then administered, with no improvement after the treatment. On the same day, the patient went to the dermatology emergency room of a local hospital, where ciprofloxacin, methylprednisolone, and sildenafil were prescribed. Despite the treatment, the patient did not notice any improvement.

**Figure 3:**
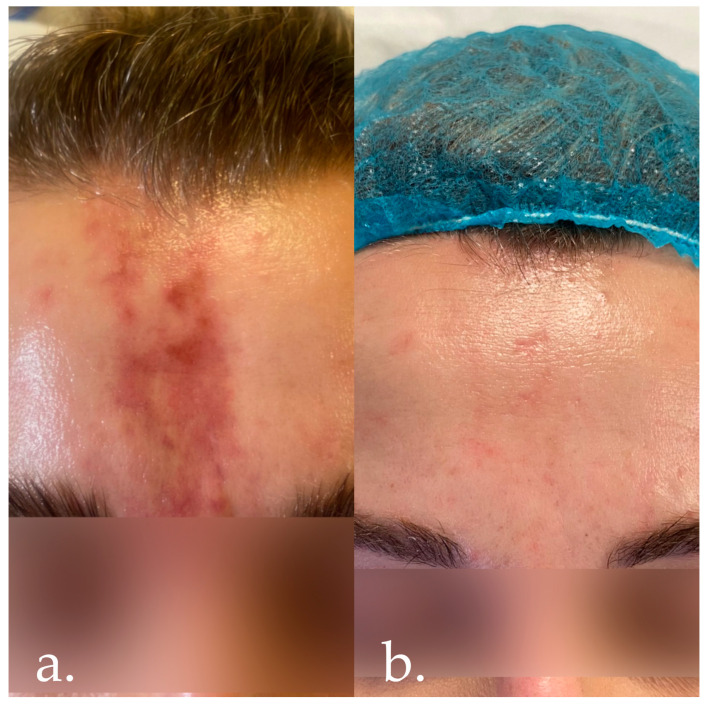
(**a**) Five days after the injection of a filler. (**b**) Three months after administration of a filler, during complementary therapy—series of three autologous platelet-rich plasma with NCTF 135 HA and two series of laser therapy using Dye-VL.

On the 5th day after the procedure (Figure 3a), the patient came to the present author’s clinic for a consultation regarding significant pain, severe swelling, softening of the tissue, and evidence of a progressive vascular compromise with clinical evidence of skin discolouration in the glabellar area. She denied any deterioration in her vision or the occurrence of other complaints. The clinician, who is also an ophthalmologist, performed a confrontational visual field test and assessed visual acuity by counting fingers from a distance of 5 m.

In the clinic, she was given dexamethasone 8 mg IM, acetylsalicylic acid 600 mg per os. and bilastine 20 mg per os. After performing an allergy test with a negative result, 1000 IU of hyaluronidase (Hylase Desau, Riemser Arzneimittel AG, Greifswald, Germany, and Esteve Pharmaceuticals GmbH, Barcelona, Spain derived from bovine testicles) was administered to the affected area and surrounding tissues, resulting in an improvement of the local condition. The patient was discharged with recommendations and general oral treatment—ciprofloxacin 500 mg (2 × 1 tab.) + probiotic, methylprednisolone 16 mg (1 × 1 tab.), bilastine 20 mg (1 × 1 tab.), acetylsalicylic acid 300 mg (2 × 1 tab.). Additionally, hot compresses were recommended for the glabellar area and surrounding tissues (3–4 times a day), and SPF50 photo-protection for the entire face.

During the follow-up visit on the following day, the patient reported persistent minor pain. The affected area began to shrink and the skin gradually regained its normal colour. Due to the presence of small areas within the glabella with questionable blood supply, 500 IU of hyaluronidase was administered preventively, which resulted in the disappearance of the above symptoms. At this stage, oral pentoxifylline 400 mg (2 × 1 tab) was added and the current general treatment was maintained for another 10 days.

At subsequent follow-up visits, the patient showed further improvement in the condition of the initially ischaemic skin and a gradual reduction in erythema (Figure 2b,c), leaving only residual redness in the projection of the earlier lesion. The absence of obstructions in blood flow in larger vessels and their branches in the frontalis and glabellar areas, as well as the formation of additional collateral vessels, was confirmed by Doppler ultrasound.

After approximately 1.5 months, in the glabellar area, a centrally located small tissue defect was noticed, which qualified for complementary treatment based on autologous, platelet-rich plasma procedures to thicken the tissue, as well as for laser therapy to remove residual erythema.

Some 2–3 months after the end of the pharmacological treatment, a series of three autologous platelet-rich plasma treatments were performed in combination with free hyaluronic acid, vitamins, and minerals (Fillmed NCTF 135 HA, LABORATORIES FILLMED, Paris, France) and two series of laser therapy using Dye-VL technology, thus achieving a satisfactorily aesthetic result (Figure 2d and Figure 3b).

## 3. Discussion

Complications of a vascular nature after the use of non-permanent tissue fillers may already have occurred during the aesthetic procedure or up to several hours thereafter, which, in the literature is defined as Nicolau Syndrome. Its characteristics include the presence of sudden severe soreness, followed by progressive swelling, skin discolouration, congestion, and tissue necrosis in the injection area. In the literature, most of the cases described concern deferred reactions of approximately 2–3 days after the procedure, which indicates the need to educate the patient regarding self-monitoring and the early recognition of possible side effects of the aesthetic procedure and also draws particular attention to the need to stay in contact and set a follow-up appointment for the patient within 7–10 days after the procedure [10,11,12].

There are three hypotheses for the mechanism of ischaemia, the first of which concerns the complete or partial occlusion of the vessel lumen with the filler as embolic material and the possible migration of acidic particles in the vessel lumen to other structures, which may consequently result in distant complications, i.e., blindness. The second hypothesis relates to vascular compression resulting from excessive pressure and pressure of the filler on the vessel from outside. The third hypothesis involves vasoconstriction due to excessive muscular tension in the vessel walls in response to a mechanical stimulus such as the supply of filler [2,3,4,5,7].

In the case described here, the potential hypothesis of ischaemia is a mixed mechanism involving the closure of the vessel lumen with a filler, along with simultaneous compression of the vessel, most likely the supratrochlear artery and its branches, as well as the supraorbital artery, due to the blanching of fine wrinkles in the forehead area.

To minimise the risk of complications, it is advisable to perform treatments with a 25G or larger cannula. The preparation should be administered slowly, in small doses, preferably using the linear retrograde technique, observing the skin as well as the patient’s subjective sensations [1,2,9]. There is now growing awareness and knowledge of the use of Doppler ultrasound in mapping the vascularisation of facial danger zones, which allows the targeted and safe administration of the filler under control.

Hyaluronidase is a glycosidase enzyme that breaks down hyaluronic acid. It is an essential part of the treatment of complications after HA acid-based fillers and is an ‘off-label’ procedure in aesthetic medicine, approved by the American Academy of Food and Drug Administration. Administration of the substance should be preceded by a sensitisation test, especially with non-human formulations, due to the risk of anaphylactic shock; however, due to the repeatedly fulminant course of vascular complications, it should be administered immediately in large quantities, according to the guidelines, upon detection of heraldic symptoms. Enzyme titration, together with complementary general pharmacology is described in the guidelines of the Polish Dermatological Society [1,2,13,14,15].

If there is an insufficient response to the enzyme administration, steroids may be added to the therapy. Corticosteroids are used to reduce inflammation and swelling and to manage immune responses associated with filler complications. It can be administered orally, intramuscularly, intravenously, or intralesionally to the area where the problem occurs; it is often employed in cases of inflammatory nodules or granulomas that may not respond adequately to hyaluronidase alone. Combining hyaluronidase and corticosteroids can be beneficial in addressing both the physical presence of the filler and the accompanying inflammatory response, ensuring a comprehensive approach to treatment.

In this article, special attention has been drawn to the need to educate doctors in the field of detecting complications after aesthetic medicine procedures, including vascular complications, which, if treated at an early enough stage, may undergo complete regression, leaving no permanent changes of either an aesthetic nature (i.e., scars, erythema), as well as the patient’s health (loss of vision, stroke).

It is worth noting that aesthetic medicine procedures are invasive procedures that directly affect the health and quality of life of patients. They are performed repeatedly in difficult areas of the face and body, so they should be performed by medical professionals (e.g., doctors) who have the necessary knowledge about the anatomy and physiology of the human body and are also authorised to start appropriate treatment at an early stage in the event of side effects [12].

## 4. Conclusions

In conclusion, this case suggested that complications after aesthetic medicine procedures, including vascular complications are possible. The education of patients and doctors, proper diagnosis, and initiation of appropriate treatment at an early stage of the problem can bring very good therapeutic results for patients.

## Data Availability

Data is available upon request to correspondence author.
